# Adipocyte STAT5 deficiency promotes adiposity and impairs lipid mobilisation in mice

**DOI:** 10.1007/s00125-016-4152-8

**Published:** 2016-11-17

**Authors:** Doris Kaltenecker, Kristina M. Mueller, Pia Benedikt, Ursula Feiler, Madeleine Themanns, Michaela Schlederer, Lukas Kenner, Martina Schweiger, Guenter Haemmerle, Richard Moriggl

**Affiliations:** 1 Ludwig Boltzmann Institute for Cancer Research, Vienna, Austria; 2 Institute of Animal Breeding and Genetics, University of Veterinary Medicine Vienna, Veterinärplatz 1, 1210 Vienna, Austria; 3 Institute of Molecular Biosciences, University of Graz, Graz, Austria; 4 Clinical Institute of Pathology, Medical University of Vienna, Vienna, Austria; 5 Unit of Pathology of Laboratory Animals, University of Veterinary Medicine Vienna, Vienna, Austria; 6 Medical University of Vienna, Vienna, Austria

**Keywords:** Adipose tissue, Energy metabolism, Lipolysis, STAT5

## Abstract

**Aims/hypothesis:**

Dysfunction of lipid metabolism in white adipose tissue can substantially interfere with health and quality of life, for example in obesity and associated metabolic diseases. Therefore, it is important to characterise pathways that regulate lipid handling in adipocytes and determine how they affect metabolic homeostasis. Components of the Janus kinase (JAK)–signal transducer and activator of transcription (STAT) pathway are involved in adipocyte physiology and pathophysiology. However, the exact physiological importance of the STAT family member STAT5 in white adipose tissue is yet to be determined. Here, we aimed to delineate adipocyte STAT5 functions in the context of lipid metabolism in white adipose tissue.

**Methods:**

We generated an adipocyte specific knockout of *Stat5* in mice using the *Adipoq*-Cre recombinase transgene followed by in vivo and in vitro biochemical and molecular studies.

**Results:**

Adipocyte-specific deletion of *Stat5* resulted in increased adiposity, while insulin resistance and gluconeogenic capacity was decreased, indicating that glucose metabolism can be improved by interfering with adipose STAT5 function. Basal lipolysis and fasting-induced lipid mobilisation were diminished upon STAT5 deficiency, which coincided with reduced levels of the rate-limiting lipase of triacylglycerol hydrolysis, adipose triglyceride lipase (ATGL, encoded by *Pnpla2*) and its coactivator comparative gene identification 58 (CGI-58). In a mechanistic analysis, we identified a functional STAT5 response element within the *Pnpla2* promoter, indicating that *Pnpla2* is transcriptionally regulated by STAT5.

**Conclusions/interpretation:**

Our findings reveal an essential role for STAT5 in maintaining lipid homeostasis in white adipose tissue and provide a rationale for future studies into the potential of STAT5 manipulation to improve outcomes in metabolic diseases.

**Electronic supplementary material:**

The online version of this article (doi:10.1007/s00125-016-4152-8) contains peer-reviewed but unedited supplementary material, which is available to authorised users.

## Introduction

White adipose tissue (WAT) has crucial functions in maintaining whole body energy homeostasis, and its unique lipid storage capacity helps to prevent ectopic lipid deposition and lipotoxicity [1, 2]. At times of energy surplus, NEFAs are deposited as triacylglycerols (TGs) in adipocytes for storage within cellular lipid droplets. Conversely, when energy becomes scarce, TGs are hydrolysed in a tightly controlled process known as lipolysis [2, 3]. Disrupting the delicate balance between lipid storage and mobilisation results in dysfunctional WAT and abnormalities in systemic lipid partitioning, which form the basis of metabolic diseases, for example in obesity and lipodystrophy [1, 4]. Since the storage and release of lipids are critical determinants of WAT integrity and organismal energy homeostasis, these processes are under tight regulation by the central nervous system as well as by counter-regulatory hormones [1, 2]. While insulin promotes TG storage, catecholamines induce lipolysis through β-adrenergic activation of protein kinase A (PKA). PKA promotes lipolysis by phosphorylating hormone-sensitive lipase (HSL; encoded by *Lipe*) and indirect activation of adipose triacylglycerol lipase (ATGL; encoded by *Pnpla2*) by phosphorylating the lipid droplet coating protein perilipin 1. Subsequently, perilipin 1 releases comparative gene identification-58 (CGI-58) from its interaction at the lipid droplet surface, thereby enabling CGI-58 to act as ATGL coactivator [5]. Although the hormonal control of lipase activation is well understood, less is known about how lipid mobilisation is transcriptionally controlled in adipocytes.

Transcription factors and associated co-regulators operate in concert to regulate metabolic pathways by modulating target gene expression via integrating endocrine, paracrine and metabolic signals [6–8]. Components of the Janus kinase (JAK)–signal transducer and activator of transcription (STAT) pathway contribute to adipocyte physiology and the pathophysiology of dysfunctional adipose tissue [9–14]. STAT5A and STAT5B (collectively referred to as STAT5) are components of the JAK–STAT pathway that are involved in adipocyte development [13, 15, 16]. However, the functions regulated by STAT5 in mature adipocytes and their consequences in vivo are largely unknown. Adipose STAT5 is prominently activated by growth hormone (GH) via JAK2 [13]. GH induces lipolysis in WAT [17, 18]; circulating levels of GH negatively correlate with WAT mass [19, 20] and adipocyte-specific deletion of *Jak2* consistently blocks GH-induced lipolysis [14, 21]. GH deficiency or defective GH receptors (GHRs) result in increased adiposity; in line with these findings, GH levels are frequently reduced in common obesity [18, 19]. Although these observations implicate STAT5 in WAT lipid metabolism, the functional importance of adipocyte STAT5 has not been genetically defined, nor have its downstream molecular mechanisms been determined.

Here, we investigated the role of STAT5 in WAT lipid homeostasis using an adipocyte-specific gene knockout in mice. To prevent interference with adipogenesis due to STAT5 deficiency, we used the *Adipoq*–Cre transgenic mice to restrict *Stat5* deletion to mature adipocytes [22].

## Methods

### Animal experiments

Adipocyte-specific STAT5-deficient mice (*Stat5* ^*Adipoq*^) were generated by crossing *Adipoq*–Cre [8] to *Stat5a/b* floxed mice [23]. Mice were housed under standardised conditions (12 h dark/12 h light cycle). Unless stated otherwise, experiments used 2-month-old male *Stat5* ^*Adipoq*^ mice or *Adipoq*–Cre negative littermates (controls; C57BL/6 background) fed a standard diet ad libitum. Animal experimentation was approved by the institutional ethics and animal welfare committee and the national authority according to Section 26 of the Animal Experiments Act (Tierversuchsgesetz 2012). Body composition was determined using an EchoMRI-100H system (EchoMRI, Houston, TX, USA). For insulin tolerance tests (ITTs), 0.75 U/kg insulin was i.p. injected into 4 h fasted mice. For OGTTs and pyruvate tolerance tests (PTTs), 1 g/kg glucose was administered via oral gavage or 2 g/kg pyruvate was i.p. injected into overnight fasted mice. The HOMA-IR was calculated from insulin and glucose levels in 4 h fasted mice: (fasting glucose [mmol/l] × fasting insulin [pmol/l]) ÷ 135. Acute β-adrenergic stimulation was performed by i.p. injection of 1 mg/kg CL-316243. Blood was collected 20 min after injection.

### Histological analysis

Formaldehyde-fixed tissues were dehydrated, paraffin-embedded, sliced, stained with haematoxylin and eosin using standard procedures and analysed by light microscopy. Adipocyte sizes were quantified from at least four different fields per mouse using ImageJ (Bethesda, MD, USA). STAT5 staining was performed as previously described [24].

### Metabolite measurements

Blood glucose and β-ketones were measured directly from tail vein blood using a glucometer (Abbott, Chicago, IL, USA). All other metabolites were measured with commercial colorimetric assays or ELISA (listed in electronic supplementary material [ESM] Methods, Metabolite measurements).

### Quantitative real-time PCR and western blotting

For RNA extraction, the RNeasy Lipid Tissue Mini Kit (Qiagen, Venlo, Netherlands) was used for tissue samples and Trizol (Thermo Fisher Scientific, Waltham, MA, USA) for 3T3-L1 adipocyte samples. RNA was reverse transcribed and cDNA was subjected to quantitative real-time PCR (qPCR) using the CFX96 Real-Time System (BioRad, Hercules, CA, USA). Samples were run in duplicate. Primers are listed in ESM Table 1. Protein extraction and western blots (40 μg protein samples) were performed as previously described [25]; antibody suppliers are listed in ESM Methods, Western blotting.

### Ex vivo measurement of lipolysis

Lipolysis of WAT explants was measured as previously described [26]. Explants were stimulated with 10 μmol/l isoprenaline or 500 ng/ml GH.

### Cell culture and adipocyte isolation

Differentiated 3T3-L1 adipocytes were incubated with GH (500 ng/ml) and/or an equimolar concentration of insulin for 6 h. For luciferase reporter assays, transfections were performed with Lipofectamine 2000 (Thermo Fisher Scientific). Cells were lysed in passive lysis buffer and luciferase activities were measured using the Dual Luciferase Reporter Assay system (Promega, Fitchburg, WI, USA). The generation of reporter constructs is described in ESM Methods, Cell culture and adipocyte isolation. Adipocyte isolation was performed using standard procedures and is described in ESM Methods, Cell culture and adipocyte isolation.

### Electrophoretic mobility shift assay and chromatin immunoprecipitation

Electrophoretic mobility shift assay [25] and chromatin immunoprecipitation (ChIP) [27] were performed as previously described. Detailed description is available in ESM Methods, Electrophoretic mobility shift assay and chromatin immunoprecipitation.

### Statistical analysis

Results are presented as means ± SEM. Two-tailed Student’s *t* tests and Wilcoxon rank-sum tests were used for comparing two groups and one-way ANOVA followed by Tukey’s honestly significant difference, Dunn’s multiple comparison or Bonferroni post hoc tests were used for comparing multiple groups. Tolerance tests were analysed with repeated measures two-way ANOVA followed by Bonferroni post hoc testing. Levels of statistical significance were set at *p < 0.05, **p < 0.01 and ***p < 0.001.

## Results

### Adipose STAT5 deficiency results in increased adiposity and reduced basal lipolysis

**Fig. 1:**
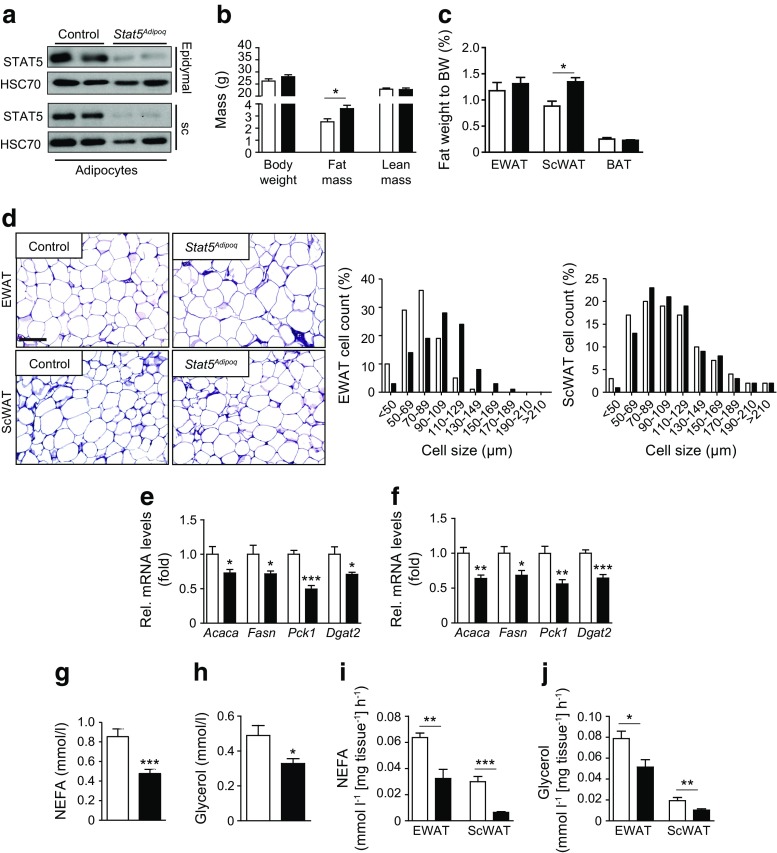
Adipocyte STAT5 deficiency promotes increased adiposity and decreased basal lipolysis. (**a**) Western blotting of isolated epididymal and subcutaneous (sc) white adipocyte protein lysates. (**b**) Body composition analysis (*n* ≥ 8). (**c**) Fat depot weight in relation to body weight (BW) (*n* ≥ 7). (**d**) Haematoxylin and eosin staining of WAT. Scale bar, 100 μm. Cell size was quantified using ImageJ (*n* ≥ 6). (**e**, **f**) Relative mRNA content for genes involved in lipid synthesis in (**e**) EWAT and (**f**) ScWAT. C_t_ values were normalised to *Gapdh* mRNA levels (*n* ≥ 5). (**g**) Plasma NEFA and (**h**) glycerol levels (*n* ≥ 6). (**i**) Basal lipolysis as indicated by NEFA and (**j**) glycerol release from WAT explants (*n* ≥ 6). *p < 0.05, **p < 0.01, ***p < 0.001. White bars, control; black bars, *Stat5* ^*Adipoq*^. Rel, relative

Immunostaining showed STAT5 levels were significantly reduced in epididymal WAT (EWAT), subcutaneous WAT (ScWAT) and interscapular brown adipose tissue (BAT) of *Stat5* ^*Adipoq*^ mice, but were unaffected in the liver (ESM Fig. 1a). Western blot analysis confirmed that STAT5 was barely detectable in extracts of isolated white adipocytes and in BAT from *Stat5* ^*Adipoq*^ mice (Fig. 1a, ESM Fig. 1b). Eight-week-old male *Stat5* ^*Adipoq*^ mice had a greater total fat mass compared with littermate controls, while their lean mass and body weight were unaltered (Fig. 1b). EWAT, BAT, liver and gastrocnemius muscle weight was unchanged, but ScWAT weight was significantly increased (Fig. 1c, ESM Fig. 1c, d). *Stat5* ^*Adipoq*^ EWAT contained a higher proportion of large adipocytes (Fig. 1d). Conversely, subcutaneous adipocytes from knockout and control animals were similar in size, suggesting that the higher amount of ScWAT in *Stat5* ^*Adipoq*^ mice was due to increased adipocyte numbers. However, levels of the adipocyte differentiation marker proteins peroxisome proliferator-activated receptor γ (PPARγ) and CCAAT/enhancer binding proteins (C/EBP) α and δ in ScWAT were similar between genotypes (ESM Fig. 1e, f). C/EBPα content was increased only in EWAT. In contrast, mRNA levels for genes involved in fatty acid (FA) and TG synthesis, such as acetyl-CoA carboxylase (*Acaca*), fatty acid synthase (*Fasn*), PEPCK (*Pck1*) and diacylglycerol O-acyltransferase 2 (*Dgat2*), were decreased in both WAT compartments of *Stat5* ^*Adipoq*^ mice (Fig. 1e, f). An increase in neither total STAT3 levels nor phosphorylation status was detectable in *Stat5* ^*Adipoq*^ WAT, suggesting that a compensatory increase in STAT3 function does not occur in response to adipose STAT5-deficiency (ESM Fig. 1g). In addition, neither typical inflammatory mRNA marker levels in WAT nor plasma IL-6 and TNF-α levels were significantly different between genotypes (ESM Fig. 1h–k). Furthermore, macrophage-related transcripts were not differentially expressed in WAT. Although not significant, plasma leptin concentration was slightly increased, while adiponectin concentration was mildly decreased (ESM Fig. 1l, m). GH levels were similar between genotypes, as were plasma TG and blood β–ketone levels (ESM Fig. 1n–p). Notably, circulating NEFA and glycerol levels were markedly decreased, indicating reduced lipid mobilisation in *Stat5* ^*Adipoq*^ mice (Fig. 1g, h). Consistent with this, basal lipolysis (determined by NEFA and glycerol release from WAT explants of fed mice) was reduced upon STAT5 deficiency (Fig. 1i, j). Therefore, our results show that adipose STAT5 deficiency increases body fat mass, which coincides with a decrease in basal lipolysis.

### Improved variables of glucose metabolism in *Stat5*^*Adipoq*^ mice

**Fig. 2:**
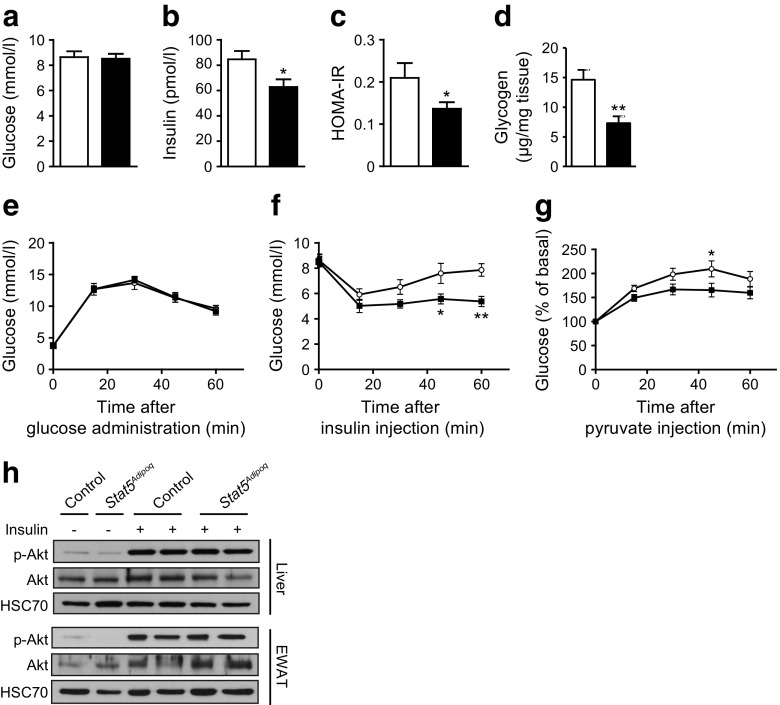
Variables of glucose metabolism are improved in *Stat5* ^*Adipoq*^ mice. (**a**) Blood glucose and (**b**) plasma insulin levels in 4 h fasted mice (*n* ≥ 6). (**c**) HOMA-IR was calculated from glucose and insulin levels of 4 h fasted mice (*n* ≥ 6). (**d**) Liver glycogen content in fed mice (*n* ≥ 6). (**e**) OGTT: 1 g/kg glucose was administered via oral gavage in 16 h fasted mice. (**f**) ITT: 0.75 U/kg insulin was i.p. injected into 4 h fasted mice. (**g**) PTT: 2 g/kg pyruvate was i.p. injected into 16 h fasted mice (*n* ≥ 7). (**h**) Western blotting of insulin-stimulated (0.75 U/kg body weight) Akt phosphorylation in liver and EWAT lysates. *p < 0.05; **p < 0.01. White bars and circles, control; black bars and squares, *Stat5* ^*Adipoq*^

Given the interdependence of lipid and glucose metabolism, we next addressed the impact of adipose STAT5 deficiency on systemic glucose homeostasis. Four h fasted *Stat5* ^*Adipoq*^ and control mice had similar blood glucose levels, despite *Stat5* ^*Adipoq*^ mice having lower insulin levels, consistent with their lower HOMA-IR (Fig. 2a–c). Liver glycogen stores were reduced in *Stat5* ^*Adipoq*^ mice (Fig. 2d), suggesting that either glycogenolysis is increased or glycogen synthesis is reduced to maintain normoglycaemia. OGTTs revealed no difference in glucose clearance between *Stat5* ^*Adipoq*^ and control littermates (Fig. 2e). However, post-hypoglycaemic recovery in *Stat5* ^*Adipoq*^ mice during ITTs was delayed: glucose levels reached only 60% of starting values at the end of the experiment (Fig. 2f). As this observation suggests a reduction in counter-regulatory mechanisms such as hepatic glucose production, we performed PTTs to measure hepatic gluconeogenesis indirectly. In agreement with ITT findings, glucose production from pyruvate was reduced in *Stat5* ^*Adipoq*^ mice (Fig. 2g). Interestingly, activating Akt S473 phosphorylation upon insulin injection was similar in liver and WAT lysates from both genotypes (Fig. 2h).

**Fig. 3:**
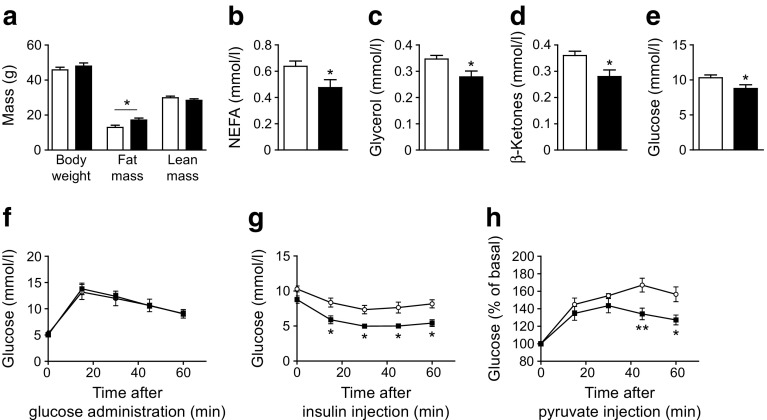
Improved insulin sensitivity in aged *Stat5* ^*Adipoq*^ mice. (**a**) Body composition analysis (*n* = 5), (**b**) plasma NEFA and (**c**) glycerol (*n* ≥ 7), (**d**) blood β-ketone and (**e**) glucose levels in 4 h fasted 52-week-old mice (*n* = 10). (**f**) OGTT: 1 g/kg glucose was administered via oral gavage in 16 h fasted mice. (**g**) ITT: 0.75 U/kg insulin was i.p. injected into 4 h fasted mice. (**h**) PTT: 2 g/kg pyruvate was i.p. injected into 16 h fasted mice (*n* ≥ 4). All tolerance tests were performed in 52-week-old mice. *p < 0.05, **p < 0.01. White bars and circles, control; black bars and squares, *Stat5* ^*Adipoq*^

As ageing is linked to deterioration in glucose metabolism, we investigated whether STAT5 deficiency also has beneficial metabolic effects at older ages. Similar to young mice, total fat mass and ScWAT mass were increased in 52-week-old *Stat5* ^*Adipoq*^ male mice (Fig. 3a; ESM Fig. 2a). Plasma NEFA and glycerol levels remained lower and blood β-ketones were reduced in aged *Stat5* ^*Adipoq*^ mice (Fig. 3b–d). Although fasting blood glucose levels were decreased in aged *Stat5* ^*Adipoq*^ mice, glucose tolerance was similar between genotypes (Fig. 3e, f). However, the increased insulin sensitivity was preserved in aged *Stat5* ^*Adipoq*^ animals, as was their reduced capacity to produce glucose from pyruvate (Fig. 3g, h).

Together, these data show that adipose STAT5 deficiency results in decreased insulin resistance scores and reduced gluconeogenic capacity.

### Acute β-adrenergic induction of lipolysis is not impaired in *Stat5*^*Adipoq*^ mice

**Fig. 4:**
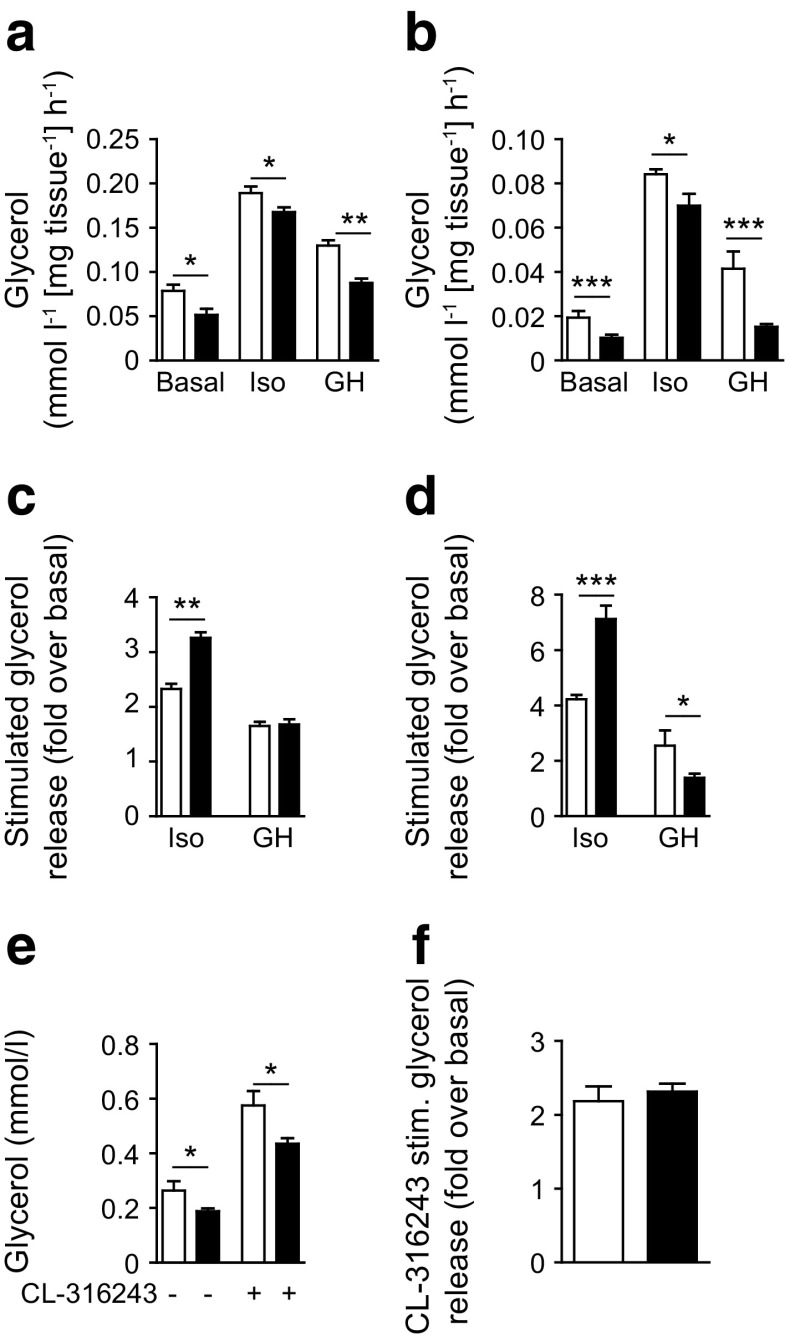
Acute β-adrenergic induction of lipolysis is not impaired in *Stat5* ^*Adipoq*^ mice. (**a**, **b**) Basal and isoprenaline (Iso)- and GH-stimulated lipolysis represented by NEFA and glycerol release from (**a**) EWAT and (**b**) ScWAT explants (*n* ≥ 5). (**c**, **d**) Fold change in glycerol levels over basal after stimulation of (**c**) EWAT and (**d**) ScWAT explants. (**e**) CL-316243-stimulated glycerol levels in plasma (*n* ≥ 5). (**f**) Fold change in glycerol levels over basal after CL-316243 treatment (*n* ≥ 5). *p < 0.05, **p < 0.01, ***p < 0.001. White bars, control; black bars, *Stat5* ^*Adipoq*^. Stim, stimulated

Our results demonstrated that basal lipolytic capacity was reduced upon STAT5 deficiency. We next determined the lipolytic response to acute β-adrenergic stimulation by treating WAT explants with isoprenaline. NEFA and glycerol levels in media from isoprenaline-stimulated *Stat5* ^*Adipoq*^ WAT explants were reduced (Fig. 4a, b, ESM Fig. 3a, b), while the fold induction of isoprenaline-stimulated lipolysis over baseline was increased (Fig. 4c, d). Lipolytic products were also reduced upon GH stimulation of *Stat5* ^*Adipoq*^ WAT explants. However, GH-mediated induction of lipolysis was only significantly decreased in STAT5-deficient ScWAT explants (Fig. 4a–d). To determine the responses to acute β-adrenergic stimulation in vivo, we treated mice with CL-316243 (a β_3_-adrenergic agonist). Although the fold induction of lipolysis was similar among genotypes, *Stat5* ^*Adipoq*^ mice displayed a ∼25% decrease in plasma glycerol and NEFA levels upon CL-316243 treatment (Fig. 4e, f, ESM Fig. 3c). These findings indicate that β-adrenergic responsiveness in *Stat5* ^*Adipoq*^ mice is intact, although the resulting induction rate is not sufficient to restore circulating lipolytic products to control levels.

### Adipocyte-specific STAT5 deficiency impairs fasting-induced lipid mobilisation

**Fig. 5:**
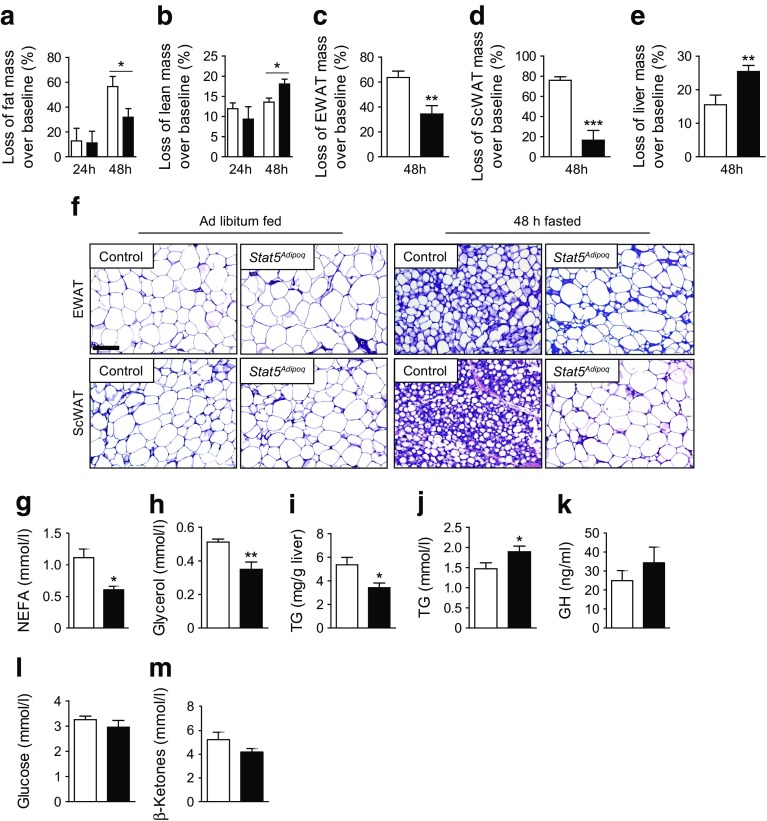
Impaired lipid mobilisation during fasting in *Stat5* ^*Adipoq*^ mice. (**a**, **b**) Loss of (**a**) total fat and (**b**) lean mass over fed state (baseline) after fasting (*n* ≥ 6). (**c**–**e**) Loss of WAT and liver mass over baseline after fasting (*n* ≥ 6). (**f**) Haematoxylin and eosin staining of WAT at baseline and after fasting (staining of WAT at baseline is depicted again here [as well as in Fig. 1d] for the purpose of comparison). Scale bar, 100 μm. (**g**) Plasma NEFA and (**h**) glycerol levels in 48 h fasted mice (*n* ≥ 6). (**i**) Liver triacylglycerol content in 48 h fasted mice. (**j**–**m**) Blood and plasma metabolite and cytokine levels in 48 h fasted mice. (*n* ≥ 6). *p < 0.05, **p < 0.01, ***p < 0.001. White bars, control; black bars, *Stat5* ^*Adipoq*^

Given the inability of *Stat5* ^*Adipoq*^ mice to compensate for defective basal release of lipolytic products upon acute β-adrenergic stimulation, we determined their ability to respond to a situation that requires lipolysis by subjecting them to a 48 h fast. There was no difference in overall body weight loss between fasted *Stat5* ^*Adipoq*^ and control mice (ESM Fig. 4a). However, *Stat5* ^*Adipoq*^ mice lost less fat mass and more lean mass during this intervention (Fig. 5a, b, ESM Fig. 4b, c). Accordingly, loss of WAT mass was decreased and loss of liver mass was increased in fasted *Stat5* ^*Adipoq*^ mice, while no changes were observed in gastrocnemius muscle and BAT weight (Fig. 5c–e, ESM Fig. 4d–h). Their inefficient lipid mobilisation was further supported by WAT histological analysis (Fig. 5f): control adipocytes displayed drastic cell shrinkage upon fasting compared with the fed state, whereas STAT5-deficient adipocytes were almost unaffected by fasting. Plasma NEFA and glycerol concentrations remained lower in 48 h fasted *Stat5* ^*Adipoq*^ mice compared with controls and fasting-associated lipid accumulation was reduced within *Stat5* ^*Adipoq*^ livers (Fig. 5g–i, ESM Fig 4i). Plasma TG levels were elevated and GH levels showed a slight but non-significant increase in fasted *Stat5* ^*Adipoq*^ mice (Fig. 5j, k). Despite the reduction in lipolytic products as substrates for energy production, fasting blood glucose and β-ketone levels were similar among genotypes (Fig. 5l, m), suggesting that catabolism of non-adipose tissue in *Stat5* ^*Adipoq*^ mice (i.e. the liver) might compensate for missing substrates that are usually provided by WAT lipid mobilisation. Taken together, our results suggest that adipose STAT5 is required not only for basal lipolysis but also for efficient lipid mobilisation under 48 h fasting conditions.

### Impaired lipolysis in *Stat5*^*Adipoq*^ mice is linked to reduced ATGL and CGI-58 levels

**Fig. 6:**
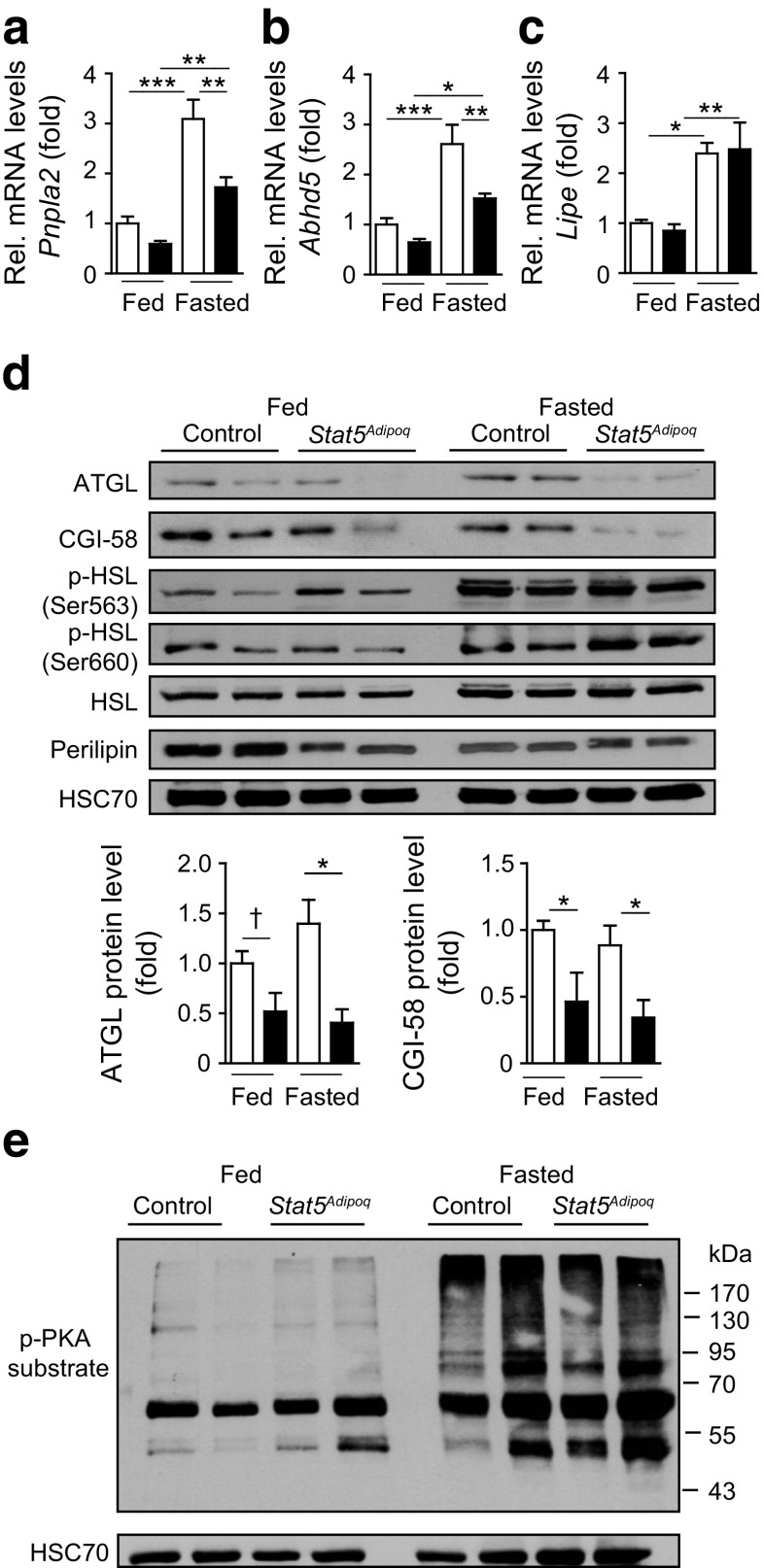
Impaired lipolysis in *Stat5* ^*Adipoq*^ mice is associated with decreased ATGL and CGI-58 levels. (**a**–**c**) EWAT mRNA levels for lipolytic genes in fed and 48 h fasted mice. C_t_ values were normalised to *Gapdh* mRNA levels (*n* ≥ 5). (**d**, **e**) Western blot of EWAT lysates shows lipolytic protein activation status and/or total levels (*n* ≥ 3). *p < 0.05, **p < 0.01; ***p < 0.001; ^†^ *p* = 0.053. White bars, control; black bars, *Stat5* ^*Adipoq*^. Rel, relative

To gain insight into the molecular changes underlying the lipolytic defect of fed and 48 h fasted *Stat5* ^*Adipoq*^ mice, we measured the mRNA levels for key lipolytic genes in EWAT. No changes were detected in fed or fasting *Lipe* (encoding HSL), *Mgll* (encoding monoacylglycerol lipase) and *Plin1* (encoding perilipin 1) mRNA levels in STAT5-deficient compared with control EWAT. In contrast, mRNA levels of *Pnpla2* (encoding ATGL) and its coactivator *Abhd5* (encoding CGI-58) were diminished in the fed state (Fig. 6a–c, ESM Fig. 5a, b). Although fasting significantly increased *Pnpla2* and *Abhd5* expression in both genotypes, levels in *Stat5* ^*Adipoq*^ mice remained significantly lower, indicating that the baseline defect cannot be efficiently compensated for by fasting. Notably, ATGL and CGI-58 protein levels were decreased in EWAT from fed and fasted *Stat5* ^*Adipoq*^ mice, whereas phosphorylation status and total levels of HSL as well as PKA activity were similar between the genotypes (Fig. 6d, e). Interestingly, perilipin 1 levels in the fed state were reduced, which might be a compensatory mechanism to facilitate access of lipases to the lipid droplet TG moiety. These results suggest that β-adrenergic signalling is not impaired in *Stat5* ^*Adipoq*^ mice and indicate that inefficient lipid mobilisation is probably due to reduced levels of ATGL and CGI-58.

### STAT5 controls *Pnpla2* expression in WAT

**Fig. 7:**
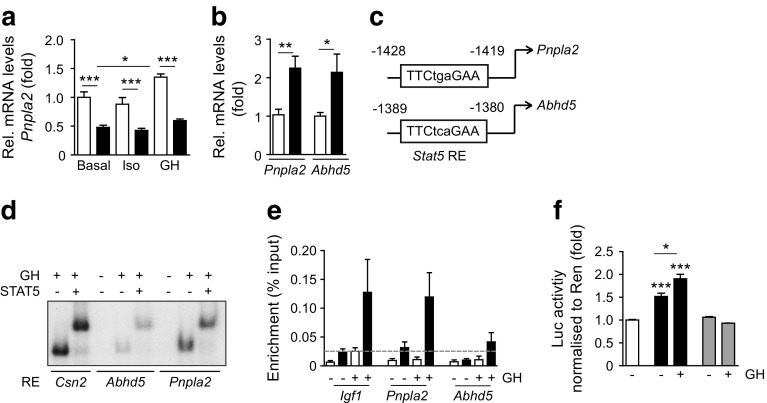
STAT5 controls *Pnpla2* expression in WAT. (**a**) *Pnpla2* mRNA levels in EWAT explants. C_t_ values were normalised to *Gapdh* mRNA levels (*n* ≥ 6). White bars, control; Black bars, *Stat5* ^*Adipoq*^. (**b**) *Pnpla2* and *Abhd5* mRNA levels in 3T3-L1 adipocytes. C_t_ values were normalised to *36B4* mRNA levels (*n* = 8). White bars, PBS; black bars, GH. (**c**) Putative *Stat5* REs in the promoter regions of *Pnpla2* and *Abhd5*. Numbers indicate the distance from translation start site. (**d**) Electrophoretic mobility shift assay. *Csn2* (encoding β-Casein) RE served as a positive control. (**e**) ChIP in EWAT. Binding of STAT5 to the *Igf1* promoter served as a positive control. The horizontal line indicates the threshold for non-specific binding (*n* ≥ 5). White bars, IgG; black bars, STAT5. (**f**) Luciferase reporter assay in NIH3T3 cells transfected with the respective reporter construct (*n* ≥ 5). *p < 0.05, **p < 0.01, ***p < 0.001. White bars, empty vector; black bars, *Pnpla2*-Luc; grey bars, *Pnpla2*-mutS5Re-luc. Luc, luciferase; Rel, relative; Ren, *Renilla*

We next performed a series of experiments to investigate the mechanisms by which STAT5 controls ATGL and CGI-58 levels. Consistent with our in vivo findings, *Pnpla2* and *Abhd5* mRNA levels in STAT5-deficient explants were reduced, while *Lipe* expression was unchanged (Fig. 7a, ESM Fig. 6a, b). After GH stimulation for 2 h, *Pnpla2* mRNA was upregulated in control explants, suggesting transcriptional activation of the *Pnpla2* gene. Consistent with this observation, GH stimulation of 3T3-L1 adipocytes significantly upregulated *Pnpla2* and restored insulin-mediated downregulation of *Pnpla2* to basal levels ([28], Fig. 7b, ESM Fig. 6c). Moreover, this treatment was sufficient to stimulate *Abhd5* upregulation in 3T3-L1 adipocytes (Fig. 7b). Consequently, we investigated whether STAT5 transcriptionally regulates *Pnpla2* and *Abhd5*. We identified a functional *Stat5* response element (RE) within the *Pnpla2* promoter region (−1428 to −1419 bp) and the *Abhd5* promoter region (−1389 to −1380 bp) by electrophoretic mobility shift assay (Fig. 7c, d). To confirm that STAT5 associates with the identified chromatin regions, we performed ChIP in EWAT extracts. Enriched binding of GH-activated STAT5 was observed on the *Pnpla2* promoter and, to a lesser extent, on the *Abhd5* promoter (Fig. 7e). To validate the functionality of the identified REs, we performed reporter assays in NIH3T3 cells in which luciferase gene expression was regulated by either a 146 bp fragment of the *Pnpla2* promoter (*Pnpla2*-luc) or a 138 bp fragment of the *Abhd5* promoter (*Abhd5*-luc) containing the respective *Stat5* RE. For *Abhd5*-luc, reporter gene activity was not induced above empty vector levels (ESM Fig. 6d); however, *Pnpla2*-luc had significantly elevated luciferase activity, which was further increased by GH stimulation (Fig. 7f). Mutation of the *Stat5* RE within the *Pnpla2* promoter fragment (*Pnpla2*-mutS5RE-luc) decreased luciferase gene expression to empty vector levels. In line with this, *Pnpla2*-luc reporter activity was abolished in *Stat5*-null mouse embryonic fibroblasts and restored by *Stat5a* co-expression (ESM Fig. 6e). These data provide mechanistic evidence that STAT5 is a transcriptional regulator of the *Pnpla2* gene in WAT.

## Discussion

We report a previously unanticipated requirement for STAT5 in maintaining lipid homeostasis in WAT. Basal lipolysis and fasting-induced lipid mobilisation were diminished by STAT5 deficiency, which was associated with decreased ATGL and CGI-58 levels. We provide further evidence that *Pnpla2* is transcriptionally regulated by STAT5 in WAT. Despite increased adiposity, a lack of STAT5 in adipocytes promoted improved insulin sensitivity and reduced gluconeogenic capacity in mice.

STAT5 deficiency differentially affected the two main WAT compartments: ScWAT mass was substantially increased despite unaltered adipocyte size, while EWAT mass was unchanged although characterised by adipocyte hypertrophy. Preferential enlargement of ScWAT has been noted upon adipocyte-specific deletion of *Jak2* using *Adipoq*–Cre [21] and in adipose *Ghr* deleted male mice using aP2-Cre [29], which may reflect different responses to GH in the respective WAT compartments [30]. Interestingly, adipose *Jak2* deletion using aP2-Cre resulted in significant enlargement of all fat depots, although ScWAT showed the greatest increase [14]. This difference may be attributable to the age, genetic background or Cre-promoter usage. A characteristic feature of both STAT5-deficient WAT compartments was a reduced capacity for basal lipolysis accompanied by decreased mRNA levels for genes involved in FA and TG synthesis (i.e. *Acaca*, *Fasn*, *Pck1* and *Dgat2*). Interestingly, chronic stimulation of lipolysis was shown to coincide with an upregulation of genes involved in FA and TG synthesis [31], while blockage of lipolysis in chow- and HFD-fed mice resulted in downregulation of these genes [32, 33]. These studies established possible interdependence between lipid mobilisation and synthesis in adipocytes and may explain the reduced expression of lipid storage related genes in STAT5-deficient WAT.

Our results indicate that β-adrenergic responsiveness in *Stat5* ^*Adipoq*^ mice is intact, although the lipolysis induction rate was not sufficient to compensate for the defective basal release of lipolytic products upon acute β-adrenergic stimulation. Similarly, the basal defect was not efficiently compensated for by fasting and thus contributed to aberrant fasting responses. On the molecular level, neither the levels nor activation status of HSL or PKA activity were altered in WAT *Stat5* ^*Adipoq*^ mice. This finding supports the observation that impaired lipid mobilisation in *Stat5* ^*Adipoq*^ mice is not due to defective β-adrenergic signalling and argues against STAT5-dependent regulation of HSL levels in WAT. Adipose STAT5 deficiency resulted in reduced ATGL and CGI-58 levels, thereby impacting the lipolytic machinery at other crucial nodes. In contrast to post-translational regulation of ATGL activity by CGI-58 and G0S2 [3, 34], little is known about the direct transcriptional regulators of *Pnpla2*: feeding and insulin both repress *Pnpla2* expression via the FoxO1 transcription factor, whereas fasting and PPARγ agonists elevate *Pnpla2* mRNA levels [2, 3, 28, 35]. Our study extends this knowledge by providing several lines of evidence supporting *Pnpla2* regulation by adipose STAT5: (1) *Pnpla2* expression is significantly increased by GH only in control WAT explants; (2) STAT5 is enriched at the *Pnpla2* promoter in WAT; and (3) a *Pnpla2* promoter fragment containing the *Stat5* RE activates a reporter gene. The presence of defects in basal lipolysis in *Stat5* ^*Adipoq*^ mice raises the possibility that STAT5 is implicated in balancing lipid metabolism in WAT. This effect may involve pulsatile GH secretion and/or its counter-regulatory effects on insulin function [18] to maintain baseline levels of ATGL and lipid turnover. In support of this notion, GH treatment of 3T3-L1 adipocytes restored insulin-mediated downregulation of *Pnpla2* to basal levels. At this point, we cannot exclude the possibility that additional mechanisms contribute, directly or indirectly, to impaired lipolysis in *Stat5* ^*Adipoq*^ mice. Given that ATGL activity requires CGI-58 [3], it is conceivable that defective lipolysis results from the simultaneous decrease in the levels of both proteins. However, as we could not show that the *Abhd5* promoter fragment has transactivation potential in reporter assays, further studies are needed to precisely define the underlying molecular mechanism.

STAT5 deficiency in WAT improved variables of glucose metabolism. Adipose tissue has a central role in glucose homeostasis and the lipolytic capacity of WAT negatively correlates with insulin sensitivity [1, 2, 32, 33]. Consistent with this, *Stat5* ^*Adipoq*^ mice displayed reduced insulin resistance scores and improved insulin sensitivity in ITTs. These results contrast with those of adipocyte *Jak2* deletion, which does not alter glucose metabolism in young mice but leads to insulin resistance accompanied by increased circulating NEFA in aged animals [14]. Despite the ability of *Stat5* ^*Adipoq*^ mice to maintain euglycaemia during fasting periods, their gluconeogenic capacity in tolerance tests was reduced (i.e. post-hypoglycaemic recovery, gluconeogenesis from pyruvate). It is worth considering that glucose production can be limited by WAT-derived substrate fluxes as glycerol is a direct gluconeogenic substrate, while NEFA oxidation indirectly activates gluconeogenesis via allosteric effectors [36–38]. Hence, diminished NEFA and glycerol availability as well as the depletion of liver glycogen may contribute to the reduced glucose production of *Stat5* ^*Adipoq*^ mice.

Collectively, our results identify STAT5 as a pivotal regulator of basal lipolysis that is needed for WAT to respond efficiently to altered energy demands. Moreover, our data indicate that glucose metabolism can be improved by interfering with adipose STAT5. Thus, our study not only extends the current understanding of WAT physiology but also provides a rationale for future studies into the potential of STAT5 manipulation to improve outcomes in metabolic diseases.

## Electronic supplementary material

Below is the link to the electronic supplementary material.

ESM (PDF 900 kb)

## Abbreviations

ATGL: Adipose triglyceride lipase
BAT: Brown adipose tissue
CGI-58: Comparative gene identification-58
ChIP: Chromatin immunoprecipitation
EWAT: Epididymal white adipose tissue
FA: Fatty acid
GH: Growth hormone
GHR: Growth hormone receptor
HSL: Hormone-sensitive lipase
ITT: Insulin tolerance test
JAK: Janus kinase
PKA: Protein kinase A
PTT: Pyruvate tolerance test
RE: Response element
ScWAT: Subcutaneous white adipose tissue
STAT: Signal transducer and activator of transcription
TG: Triacylglycerols
WAT: White adipose tissue

## References

[1] Rosen ED, Spiegelman BM (2014). What we talk about when we talk about fat. Cell.

[2] Zechner R, Zimmermann R, Eichmann TO (2012). Fat signals – lipases and lipolysis in lipid metabolism and signaling. Cell Metab.

[3] Lass A, Zimmermann R, Oberer M, Zechner R (2011). Lipolysis – a highly regulated multi-enzyme complex mediates the catabolism of cellular fat stores. Prog Lipid Res.

[4] Cao H (2014). Adipocytokines in obesity and metabolic disease. J Endocrinol.

[5] Peckett AJ, Wright DC, Riddell MC (2011). The effects of glucocorticoids on adipose tissue lipid metabolism. Metabolism.

[6] Desvergne B, Michalik L, Wahli W (2006). Transcriptional regulation of metabolism. Physiol Rev.

[7] Rohm M, Sommerfeld A, Strzoda D (2013). Transcriptional cofactor TBLR1 controls lipid mobilization in white adipose tissue. Cell Metab.

[8] Eguchi J, Wang X, Yu S (2011). Transcriptional control of adipose lipid handling by IRF4. Cell Metab.

[9] Cernkovich ER, Deng J, Bond MC, Combs TP, Harp JB (2008). Adipose-specific disruption of signal transducer and activator of transcription 3 increases body weight and adiposity. Endocrinology.

[10] Derecka M, Gornicka A, Koralov SB (2012). Tyk2 and Stat3 regulate brown adipose tissue differentiation and obesity. Cell Metab.

[11] Tsoli M, Schweiger M, Vanniasinghe AS (2014). Depletion of white adipose tissue in cancer cachexia syndrome is associated with inflammatory signaling and disrupted circadian regulation. PLoS One.

[12] Moisan A, Lee YK, Zhang JD (2015). White-to-brown metabolic conversion of human adipocytes by JAK inhibition. Nat Cell Biol.

[13] Richard AJ, Stephens JM (2014). The role of JAK-STAT signaling in adipose tissue function. Biochim Biophys Acta.

[14] Shi SY, Luk CT, Brunt JJ (2014). Adipocyte-specific deficiency of Janus kinase (JAK) 2 in mice impairs lipolysis and increases body weight, and leads to insulin resistance with ageing. Diabetologia.

[15] Jung HS, Lee YJ, Kim YH, Paik S, Kim JW, Lee JW (2012). Peroxisome proliferator-activated receptor gamma/signal transducers and activators of transcription 5A pathway plays a key factor in adipogenesis of human bone marrow-derived stromal cells and 3T3-L1 preadipocytes. Stem Cells Dev.

[16] Kawai M, Namba N, Mushiake S (2007). Growth hormone stimulates adipogenesis of 3T3-L1 cells through activation of the Stat5A/5B-PPARγ pathway. J Mol Endocrinol.

[17] Moller L, Norrelund H, Jessen N (2009). Impact of growth hormone receptor blockade on substrate metabolism during fasting in healthy subjects. J Clin Endocrinol Metab.

[18] Moller N, Jorgensen JO (2009). Effects of growth hormone on glucose, lipid, and protein metabolism in human subjects. Endocr Rev.

[19] Berryman DE, Glad CA, List EO, Johannsson G (2013). The GH/IGF-1 axis in obesity: pathophysiology and therapeutic considerations. Nat Rev Endocrinol.

[20] Chaves VE, Junior FM, Bertolini GL (2013). The metabolic effects of growth hormone in adipose tissue. Endocrine.

[21] Nordstrom SM, Tran JL, Sos BC, Wagner KU, Weiss EJ (2013). Disruption of JAK2 in adipocytes impairs lipolysis and improves fatty liver in mice with elevated GH. Mol Endocrinol.

[22] Berry R, Rodeheffer MS (2013). Characterization of the adipocyte cellular lineage in vivo. Nat Cell Biol.

[23] Cui Y, Riedlinger G, Miyoshi K (2004). Inactivation of Stat5 in mouse mammary epithelium during pregnancy reveals distinct functions in cell proliferation, survival, and differentiation. Mol Cell Biol.

[24] Schlederer M, Mueller KM, Haybaeck J (2014). Reliable quantification of protein expression and cellular localization in histological sections. PLoS One.

[25] Engblom D, Kornfeld JW, Schwake L (2007). Direct glucocorticoid receptor-Stat5 interaction in hepatocytes controls body size and maturation-related gene expression. Genes Dev.

[26] Schweiger M, Eichmann TO, Taschler U, Zimmermann R, Zechner R, Lass A (2014). Measurement of lipolysis. Methods Enzymol.

[27] Grabner B, Schramek D, Mueller KM (2015). Disruption of STAT3 signalling promotes KRAS-induced lung tumorigenesis. Nat Commun.

[28] Kim JY, Tillison K, Lee JH, Rearick DA, Smas CM (2006). The adipose tissue triglyceride lipase ATGL/PNPLA2 is downregulated by insulin and TNF-α in 3T3-L1 adipocytes and is a target for transactivation by PPARγ. Am J Phys Endocrinol Metab.

[29] List EO, Berryman DE, Funk K (2013). The role of GH in adipose tissue: lessons from adipose-specific GH receptor gene-disrupted mice. Mol Endocrinol.

[30] Berryman DE, List EO, Sackmann-Sala L, Lubbers E, Munn R, Kopchick JJ (2011). Growth hormone and adipose tissue: beyond the adipocyte. Growth Horm IGF Res.

[31] Mottillo EP, Balasubramanian P, Lee YH, Weng C, Kershaw EE, Granneman JG (2014). Coupling of lipolysis and de novo lipogenesis in brown, beige, and white adipose tissues during chronic β3-adrenergic receptor activation. J Lipid Res.

[32] Li YQ, Shrestha YB, Chen M, Chanturiya T, Gavrilova O, Weinstein LS (2016). Gsalpha deficiency in adipose tissue improves glucose metabolism and insulin sensitivity without an effect on body weight. Proc Natl Acad Sci U S A.

[33] Schreiber R, Hofer P, Taschler U (2015). Hypophagia and metabolic adaptations in mice with defective ATGL-mediated lipolysis cause resistance to HFD-induced obesity. Proc Natl Acad Sci U S A.

[34] Yang X, Lu X, Lombes M (2010). The G_0_/G_1_ switch gene 2 regulates adipose lipolysis through association with adipose triglyceride lipase. Cell Metab.

[35] Chakrabarti P, Kandror KV (2009). FoxO1 controls insulin-dependent adipose triglyceride lipase (ATGL) expression and lipolysis in adipocytes. J Biol Chem.

[36] Samuel VT, Shulman GI (2016). The pathogenesis of insulin resistance: integrating signaling pathways and substrate flux. J Clin Invest.

[37] Rebrin K, Steil GM, Getty L, Bergman RN (1995). Free fatty acid as a link in the regulation of hepatic glucose output by peripheral insulin. Diabetes.

[38] Perry RJ, Zhang XM, Zhang D (2014). Leptin reverses diabetes by suppression of the hypothalamic-pituitary-adrenal axis. Nat Med.

