# Supplementary material for: Adipocyte STAT5 deficiency promotes adiposity and impairs lipid mobilisation in mice
Source: Diabetologia. 2016 Nov 17;60(2):296–305. doi: 10.1007/s00125-016-4152-8 (PMC6518368; doi:10.1007/s00125-016-4152-8)
Supplement: Supplementary file 1 — (PDF 900 kb) [file 125_2016_4152_MOESM1_ESM.pdf]

Electronic Supplementary Material (ESM)

Metabolite measurements. Plasma NEFA were determined with the NEFA-HR(2) kit (Wako Chemicals, Neuss, Germany) and glycerol with the Free Glycerol Reagent (Sigma, St. Louis, MO, USA). Plasma triglyceride concentration was measured with the Reflotron Plus analyser (Roche, Rotkreuz, Switzerland). Plasma insulin, adiponectin, leptin and GH levels were determined by ELISA (Mouse Insulin ELISA, Crystal Chem, Downers Grove, IL, USA ; Mouse Adiponectin ELISA, Mouse Leptin ELISA, Rat/Mouse Growth Hormone ELISA, all from Millipore, Billerica, MA, USA). Plasma TNFα and IL-6 were measured using the ProcartaPlex multiplex ELISA (eBioscience, San Diego, CA, USA). Total liver TG content was determined using a Triglyceride Colorimetric Assay Kit (Cayman Chemical, Ann Arbor, MI, USA); liver glycogen concentrations were measured with a Glycogen Assay Kit (Sigma).

Western blotting

Blots were incubated with antibodies against pS473-AKT (#9271), ATGL (#2439), Perilipin (#9349), pSer563-HSL (#4139), pSer660-HSL(#4126), HSL (#4107), pTyr705-STAT3 (#9131), pPKA Substrate (#9624; all from Cell Signaling, Danvers, MA, USA), HSC70 (sc-7298), CGI-58 (sc-100468), STAT5 (sc-835), AKT1/2/3 (sc-8312), PPARγ (E8, sc-7273), C/EBPδ (M17, sc-636), C/EBPα (14AA, sc-61; all from Santa Cruz Biotechnology, Santa Cruz, CA, USA) and STAT3 (BD Biosciences, Franklin Lakes, NJ, USA). Antibodies were used in a 1:1000 dilution except for HSC70, which was used 1:10.000.

Cell culture and adipocyte isolation. To prepare luciferase reporter constructs containing the *Pnpla2* promoter fragment and *Abhd5* promoter fragment with the respective STAT5 responsive element (RE), DNA obtained from BAC clones (BAC RP23-103H3 for Pnpla2 and BAC RP23-12I4 for Abhd5) was subjected to PCR by using primers listed in ESM-Table 1. For *Pnpla2*, primer sequences included a 5´XhoI site and a 3´HindIII site, while for *Abhd5*, a 5´EcoRV and 3´HindIII were used. The PCR products were ligated into the corresponding restriction sites of a pGL4.26 vector (Promega, Fitchburg, WI, USA). Promoter constructs were confirmed by sequencing. The *Pnpla2* fragment containing the mutated STAT5 RE was synthesised by a commercial provider. The wild type sequence TTCTGAGAA was mutated to T**G**CTGAG**C**A. NIH3T3 cells were seeded in a 24-well format and co-transfected with the promoter construct and pRL-SV40 vector (Promega) encoding Renilla luciferase. Cells were stimulated with GH (500 ng/ml) for 14 h 1 day post-transfection. STAT5-null mouse embryonic fibroblasts (MEFs) were co-transfected with *Pnpla2* RE-luc, pMSCV-*Stat5a* and

pRL-SV40. All transfections were carried out using Lipofectamin®2000 (Thermo Fisher Scientific). Cells were lysed in passive lysis buffer and luciferase activities were measured using the Dual Luciferase Reporter Assay system (Promega) and an EnSpire® Multimode Plate Reader (PerkinElmer, Waltham, MA, USA).

For isolation of adipocytes, freshly isolated WAT was minced into small pieces followed by digestion in DMEM supplemented with 1mg/ml collagenase II, 75 U/ml DNAse I and 1,5% BSA for 120 minutes (min) and moderate shaking (140 rpm) at 37°C. Cell suspensions were filtered through a 200 µm cell strainer and centrifuged for 5 min at 200 g. The upper adipocyte layer was transferred into a new tube, washed with PBS and protein was isolated as described.

Electrophoretic mobility shift assay and chromatin immunoprecipitation. For electrophoretic mobility shift assays, HEK293 cells overexpressing STAT5 were stimulated with GH (500 ng/ml) for 30 min. Protein was extracted in whole cell extract buffer (20 mmol/l Hepes, pH 7.9; 20% Glycerol; 50 mmol/l KCl; 1 mmol/l EDTA; 1 mmol/l DTT; 400 mmol/l NaCl; 5 µg/ml Leupeptin; 0.2 U/ml Aprotinin; 1 mmol/l PMSF; 5 mmol/l Na_3VO_4; 10 mmol/l NaF; 5 mmol/l β-glycerophosphate). Oligos were annealed and radioactively labelled with γATP P^{32}. DNA binding capacity was evaluated in whole cell extracts. Supershifts were carried out by incubating the complexes with total STAT5 antibodies. Oligos are listed in EMS-Table 1.

For chromatin immunoprecipitation (ChIP), one freshly isolated fat pad from GH (2 µg/g, 30 min) or mock injected mice was minced into ~2 mm pieces in PBS supplemented with inhibitors (1 mmol/l Na_3VO_4, 1x Complete Protease Inhibitor Cocktail (PIC), Roche) and fixed by adding formaldehyde to 1% for 11 min, rolling at 14 rpm. Fixation was quenched by incubating with glycine at a final concentration of 125 mmol/l for 5 min, 14 rpm, before centrifuging the samples (5 min, 2400 rpm). From now on, samples were kept on ice. By use of a Pasteur pipette, PBS was aspirated and the upper phase containing the fat pellet was washed twice with ice cold PBS before incubating in buffer 1 (0.25% Triton-X, 10 mmol/l EDTA, 0.5 mmol/l EGTA, 10 mmol/l HEPES) supplemented with inhibitors for 10 min. Samples were homogenised using a dounce homogeniser (~25-30 strokes). After centrifugation (4°C), fat pellets were incubated in buffer 2 (0.2 mol/l NaCl, 1 mmol/l EDTA, 0.5 mmol/l EGTA, 10 mmol/l HEPES) supplemented with inhibitors for 10 min and centrifuged one more time. Samples were sonicated in lysis buffer (1% SDS, 10 mmol/l EDTA, 50 mmol/l Tris (pH 8.1), 1 mmol/l Na_3VO_4, 1 mmol/l phenylmethylsulphonyl

fluoride, 1x PIC) using a Diagenode bioruptor (25 cycles with 30 seconds ON, 30 seconds OFF, high magnitude). 50 µg of centrifugation cleared chromatin in dilution buffer (167 mmol/l NaCl, 16.7 mmol/l Tris (pH 8.1), 1.2 mmol/l EDTA, 1.1% Triton-X, 0.01% SDS) supplemented with inhibitors were incubated rolling at 4°C with 5 µg STAT5 (sc-835 X) or IgG (sc-2027 X, both from Santa Cruz Biotechnology) for 1 h. 1% of diluted chromatin was kept as input control. 50 µl of pre-cleared beads (Life Technologies, Carlsbad, CA, USA) were added per immune precipitation and incubated rolling overnight at 4 °C. Beads were washed once in RIPA (0.15 mol/l NaCl, 2.5 mmol/l Tris (pH 8.1), 0.1% SDS, 0.5% Sodium deoxycholate, 1% NP-40), high salt (0.5 mmol/l NaCl, 2.5 mmol/l Tris (pH 8.1), 0.1% SDS, 1% NP-40), lithium chloride (0.25 mmol/l $LiCl_2$, 2.5 mmol/l Tris (pH 8.1), 0.5% Sodium deoxycholate, 1% NP-40) and twice in TE buffer. Chromatin was eluted in 400 µl elution buffer (2% SDS, 0.1 mol/l $NaHCO_3$, 10 mmol/l dithiothreitol) and heavy shaking for 30 min. Samples and input (with elution buffer added) were reverse cross-linked with 20 µl 4 mol/l NaCl at 65 °C for at least 4 hours. Samples were incubated with 0.5 mol/l EDTA, 1 mol/l Tris (pH 6.5) and Proteinase K (10 mg/ml) at 55 °C overnight. DNA was purified using Phenol/ Chloroform/Isoamyl Alcohol (Invitrogen, Life technologies). DNA was subjected to qPCR using KAPA SYBR Fast DNA Polymerase (Peqlab, Erlangen, Germany) and amount of amplification was quantified with standard curves. Oligos are listed in ESM-Table 1.

ESM-Table 1: List of oligos

Oligo	Sequence
**qPCR**	
*Gapdh*_1<br>*Gapdh*_2	AGAAGGTGGTGAAGCAGGCATC<br>CGGCATCGAAGGTGGAAGAGTG
*Lipe*_1<br>*Lipe*_2	GGAGAGAGTCTGCAGGAACG<br>CCTGCAAGAGTATGTCACGC
*Pnpla2*_1<br>*Pnpla2*_2	GCCACTCACATCTACGGAGC<br>GTTGAAGGAGGGATGCAGAG
*Mgll*_1<br>*Mgll*_2	AAAGTTTGTCGGAGAATCGG<br>TTTTCCAGAACACACCCCTG
*Plin1*_1<br>*Plin1*_2	GACACCACCTGCATGGCT<br>TGAAGCAGGGCCACTCTC
*Abdh5*_1<br>*Abdh5*_2	GATGTGGGACACCAGGTAGG<br>CGGTGATGAAAGCGATGG
*Acaca*_1<br>*Acaca*_2	GAAGCCACAGTGAAATCTCG<br>GATGGTTTGGCCTTTCACAT
*Fasn*_1<br>*Fasn*_2	TGTCTGACACTGGCAATCTGAT<br>CGGTCACACGGGTAGGTAGC
*Pparg*_1<br>*Pparg*_2	ACCCAATGGTTGCTGATTAC<br>CGGGAAGGACTTTATGTATGAG
*G6pc*_1<br>*G6pc*_2	GTGTCCAGGACCCACCAATA<br>ACTGTGGGCATCAATCTCCT
*Pck1*_1<br>*Pck1*_2	CGTTTTCTGGGTTGATAGCC<br>CCTAGTGCCTGTGGGAAGAC
*36B4*_1<br>*36B4*_2	GCTTCATTGTGGGAGCAGACA<br>CATGGTGTTCTTGCCCATCAG
*Dgat2*_1<br>*Dgat2*_2	TGGCATAAGGCCCTATTTGG<br>ATGGTGTCTCGGTTGACAGG
*Tnfa*_1<br>*Tnfa*_2	TAGCCAGGAGGGAGAACAGA<br>TTTTCTGGAGGGAGATGTGG
*Il6*_1<br>*Il6*_2	TTCCATCCAGTTGCCTTCTTGG<br>TTCTCATTTCCACGATTTCCCAG
*Emr1*_1<br>*Emr1*_2	GTCTGGGTGTCAGTGCAGG<br>GGATGTACAGATGGGGGATG
*Ccl2*_1<br>*Ccl2*_2	ATTGGGATCATCTTGCTGGT<br>CCTGCTGTTCACAGTTGCC
*Cd68*_1<br>*Cd68*_2	ACCGCCATGTAGTCCAGGTA<br>ATCCCCACCTGTCTCTCTCA
*Il10*_1<br>*Il10*_2	ACCTGCTCCACTGCCTTGCT<br>GGTTGCCAAGCCTTATCGGA
**EMSA**	
Pnpla2 RE EMSA_1<br>*Pnpla2* RE EMSA_2	CATTTTCTGAGAAAACT<br>AGTTTTCTCAGAAAATG
Abhd5 RE_1<br>*Abhd5* RE_2	CATATTCTCAGAATTTC<br>GAAATTCTGAGAATATG
Positive control *Csn2*_1	AGATTTCTAGGAATTCAAATC
Positive control *Csn2*_2	GATTTGAATTCCTAGAAATCT
**ChIP**	
Positive control *Igf1*_1	CCCAAGCTGCAGAAGAGAAA

Positive control *Igf1*_2	TGATAACAGTATGCCAACACCA
Negative region_1<br>Negative region_2	TACCCCTTCCAACTCTGACTGAGC<br>TTCCCTCCAGGATGTGACTGTG
Pnpla2 RE ChIP_1<br>*Pnpla2* RE ChIP_2	GCTCACATCCAAGGGTTTTC<br>TAAGGGGATCCTCACACGTC
Abhd5 RE ChIP_1<br>*Abhd5* RE ChIP_2	ACCCAGGTGTGGCTGAACTA<br>AGCTGGAGCTGTGTTGTGC
**Luciferase reporter constructs**	
Pnpla2 RE **XhoI**<br>*Pnpla2* RE **HindIII**	ATGC**CTCGAG**GCTCACATCCAAGGGTTTTC<br>TAAGGGGATCCTCACACGTC**AAGCTT**GCAT
Abhd5 RE **EcoRV**<br>*Abhd5* RE **HindIII**	ATGC**GATATC**ACCCAGGTGTGGCTGAACTA<br>AGCTGGAGCTGTGTTGTGC**AAGCTT**GCAT

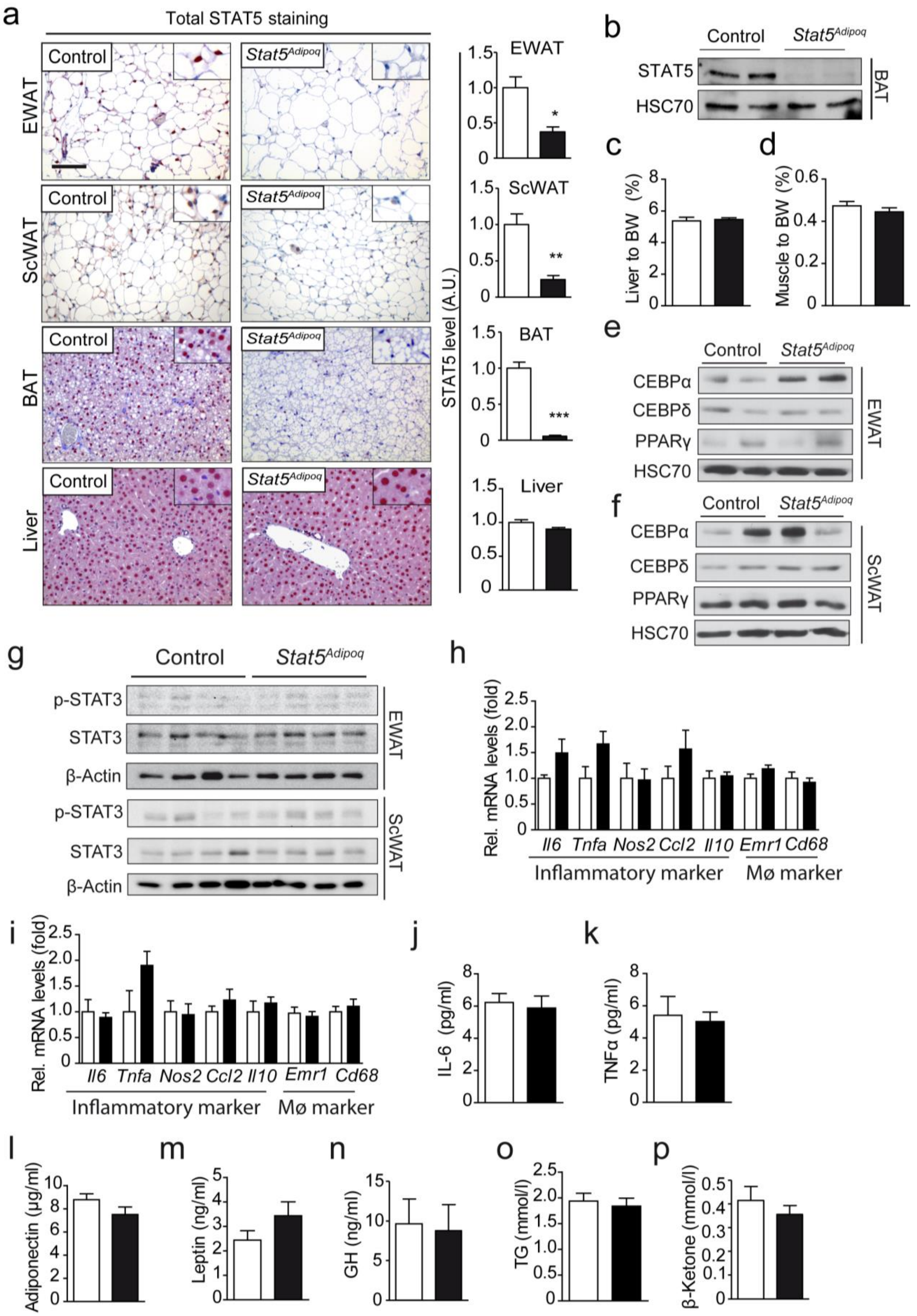

ESM-Fig. 1. STAT5 deletion efficiency and basal phenotype. (**a**) Representative paraffin sections of epididymal white adipose tissue (EWAT), subcutaneous WAT (ScWAT), brown

adipose tissue (BAT) and liver from 8-week-old mice. Sections were immunostained with STAT5. Scale bar indicates 100µm. Quantification was performed using ImageJ (n=3 mice/genotype; n≥4 fields per mouse). (**b**) Western blot of BAT lysates was performed using antibodies against STAT5. HSC70 served as loading control. (**c**) Liver and (**d**) gastrocnemius (GN) muscle weight in relation to body weight (BW) (n≥6). (**e**) Western blotting of EWAT and (**f**) ScWAT lysates was performed using antibodies against C/EBPα, C/EBPδ and PPARγ. HSC70 served as loading control. (**g**) Western blotting of EWAT lysates was performed using antibodies against p-STAT3 (Tyr705) and STAT3. HSC70 served as loading control. (**h**) Relative mRNA levels of inflammatory genes in EWAT and (**i**) ScWAT (*n*≥5). Mø: Macrophage. (**j-p**) Plasma/blood levels of cytokines, adipokines or metabolites (*n*≥6). *p<0.05, **p<0.01, ***p<0.001. White bars: control. Black bars: *Stat5^{Adipoq}*.

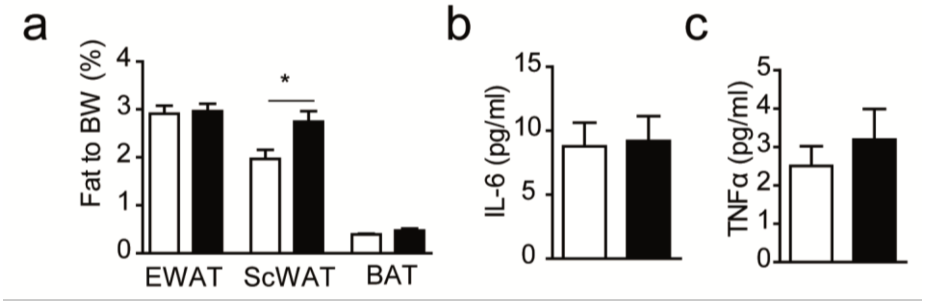

ESM-Fig. 2. Phenotype of aged *Stat5^{Adipoq}* mice. (**a**) Fat depot weight in relation to BW (*n*≥5). (**b-c**) Plasma cytokine levels (*n*≥6). Experiments were performed with 52-week-old mice. *p<0.05. White bars: control. Black bars: *Stat5^{Adipoq}*.

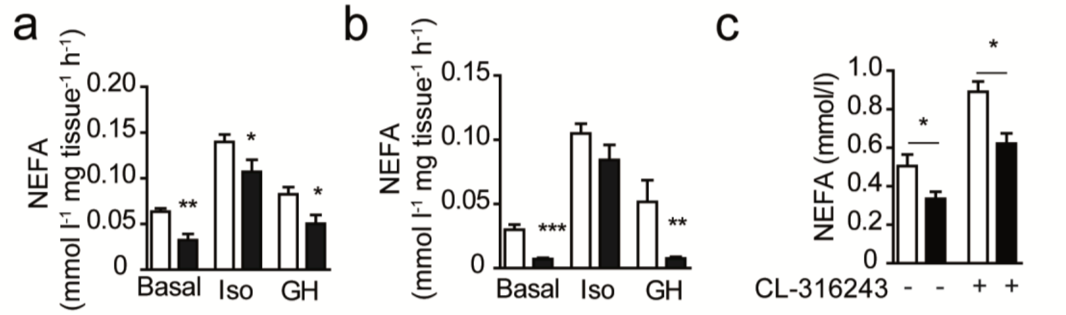

ESM-Fig. 3. (**a**) Basal (depicted for comparison), isoprenaline (Iso) and GH stimulated lipolysis of EWAT and (**b**) ScWAT explants reflected by NEFA release (*n*≥6). (**c**) CL-316243 stimulated plasma NEFA levels (*n*≥5). *p<0.05, **p<0.01, ***p<0.001. White bars: control. Black bars: *Stat5^{Adipoq}*.

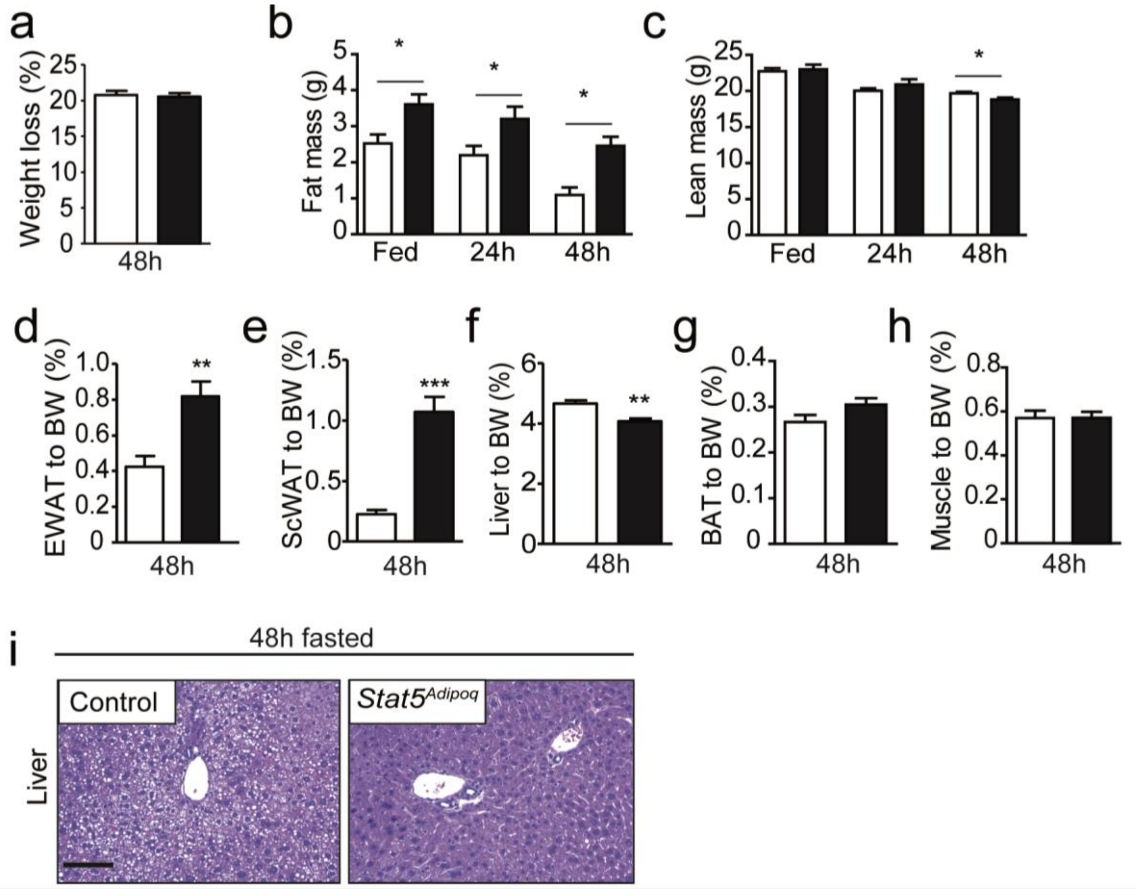

ESM-Fig. 4. Impaired lipid mobilisation capacity during fasting in *Stat5*$^{\textit{Adipoq}}$ mice. (**a**) Total BW loss after fasting. (**b**) Total fat and (**c**) lean mass in the fed state (depicted for comparison) and after prolonged fasting conditions (*n*≥6). (**d**) EWAT, (**e**) ScWAT, (**f**) liver, (**g**) BAT and (**h**) GN muscle weight in relation to BW after 48 h of fasting (*n*≥7). (**i**) Representative H&E staining of liver sections after fasting. Scale bar indicates 100 µm. **p*<0.05, ***p*<0.01, ****p*<0.001. White bars: control. Black bars: *Stat5*$^{\textit{Adipoq}}$.

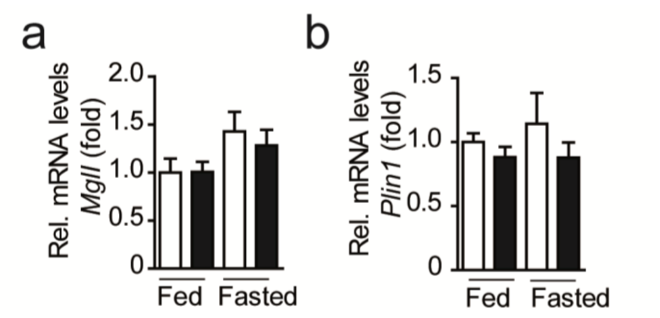

ESM-Fig. 5. Impaired lipolysis in *Stat5*$^{\textit{Adipoq}}$ mice is not associated with defective PKA signalling. (**a**) Fed and 48h-fasted mRNA levels of *MglI* and (**b**) *Plin1* in EWAT. Ct values were normalised to *Gapdh* (*n*≥5). *Mgll*: monoacylglycerol lipase; *Plin1*: perilipin 1. White bars: control. Black bars: *Stat5*$^{\textit{Adipoq}}$.

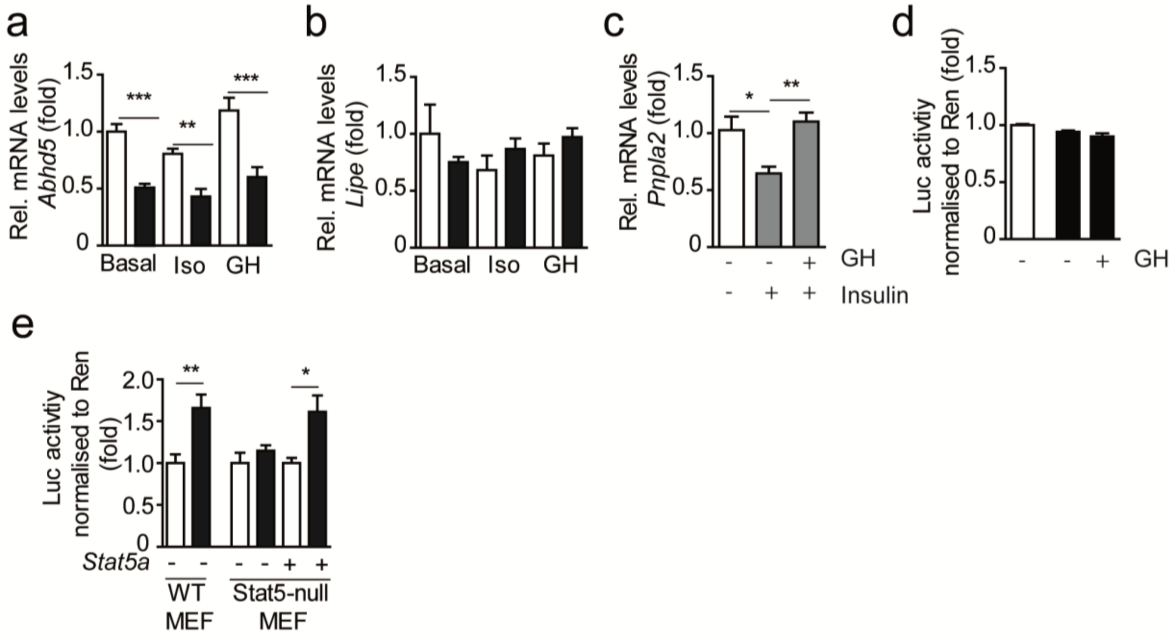

ESM-Fig. 6. STAT5 controls *Pnpla2* expression in WAT. (**a**) *Abhd5* and (**b**) *Lipe* mRNA expression of basal and stimulated lipolysis in EWAT explants. Ct values were normalised to *Gapdh* (n≥6). *Abhd5*: comparative gene identification-58; *Lipe*: hormone sensitive lipase. (**c**) Relative mRNA expression of *Pnpla2* after stimulation with insulin or co-stimulation with insulin and GH in 3T3-L1 adipocytes. Ct values were normalised to *36B4* (n≥7). (**d**) *Abhd5* response element (RE) luciferase reporter assay in NIH3T3 cells (n≥4). (**e**) *Pnpla2* RE luciferase reporter assay in wild type and Stat5-null mouse embryonic fibroblasts (MEFs) (n≥5). *p<0.05, **p<0.01, ***p<0.001. White bars: (**a,b**) control; (**c**) PBS; (**d,e**) empty vector. Black bars: (**a,b**) *Stat5*^{Adipoq}; (**d**) *Abhd5*-Luc; (**e**) *Pnpla2*-Luc.
